# Tempered *mlo* broad-spectrum resistance to barley powdery mildew in an Ethiopian landrace

**DOI:** 10.1038/srep29558

**Published:** 2016-07-12

**Authors:** Xintian Ge, Weiwei Deng, Zheng Zhou Lee, Francisco J. Lopez-Ruiz, Patrick Schweizer, Simon R. Ellwood

**Affiliations:** 1 Centre for Crop and Disease Management, Department of Environment and Agriculture, Curtin University, Bentley, WA 6102, Australia; 2 Leibniz-Institut für Pflanzengenetik und Kulturpflanzenforschung (IPK) Gatersleben, Corrensstrasse 3, Seeland, 06466 Stadt, Germany

## Abstract

Recessive mutations in the *Mlo* gene confer broad spectrum resistance in barley (*Hordeum vulgare*) to powdery mildew (*Blumeria graminis* f. sp. *hordei*), a widespread and damaging disease. However, all alleles discovered to date also display deleterious pleiotropic effects, including the naturally occurring *mlo-11* mutant which is widely deployed in Europe. Recessive resistance was discovered in Eth295, an Ethiopian landrace, which was developmentally controlled and quantitative without spontaneous cell wall appositions or extensive necrosis and loss of photosynthetic tissue. This resistance is determined by two copies of the *mlo-11* repeat units, that occur upstream to the wild-type *Mlo* gene, compared to 11–12 in commonly grown cultivars and was designated *mlo-11* (*cnv2*). *mlo-11* repeat unit copy number-dependent DNA methylation corresponded with cytological and macroscopic phenotypic differences between copy number variants. Sequence data indicated *mlo-11* (*cnv2*) formed via recombination between progenitor *mlo-11* repeat units and the 3′ end of an adjacent stowaway MITE containing region. *mlo-11* (*cnv2*) is the only example of a moderated *mlo* variant discovered to date and may have arisen by natural selection against the deleterious effects of the progenitor *mlo-11* repeat unit configuration.

Powdery mildews are a group of fungal diseases caused by obligate biotrophic species (species that derive nutrients from living tissue). Their distribution is world-wide and they infect most staple crops. The disease is characterised by white epidermal colonies composed of mycelia and spore chains. Powdery mildews stunt plant growth and hence reduce yield, while necrotic and chlorotic discoloration of surface tissue downgrades crop quality. In temperate regions barley powdery mildew, caused by *Erysiphe graminis* f. sp. *hordei* (*Bgh*), is a significant pathogen^1^.

In agriculture, the commonest form of resistance in plants to biotrophic fungi follows the race-specific gene-for-gene principle first demonstrated by Flor^2^, where recognition is governed by the direct or indirect interaction between the product of a host disease resistance gene (*R*-gene) and the complementary product of a pathogen avirulence gene^3^. While such *R*-genes are easy to select for, they are easily overcome by the pathogen as avirulence genes readily mutate, resulting in lack of recognition by a plant genotype carrying the corresponding *R*-gene. Successive rounds of wide scale monocultures leads to isolates harbouring large numbers of virulence genes, with virulence to almost all *R*-genes deployed in Europe present in just two isolates^4^.

Advances in molecular genetic techniques have renewed interest in identifying alternatives to major *R*-genes to improve crop productivity. Among these are broad-spectrum partial resistance genes. Partial resistance may be developmentally apparent or present in all growth stages and is characterised by reduced numbers of successful infection sites, slower disease progression, and fewer successful fruiting bodies. Partial resistance is common in plants but often absent in modern barley cultivars, where genetic bottlenecks and breeding between elite cultivars has led to extinction of diversity in some genomic regions and increased linkage disequilibrium^5^,^6^. Combining partial resistance genes phenotypically is challenging because of their quantitative nature but improvements in genetic marker technologies has allowed key barley QTL to be identified^4^,^7^,^8^.

Broad-spectrum resistance to powdery mildew in plants also occurs as single, naturally occurring genes with a major effect. Only two are currently known: *RPW8*, identified in Arabidopsis conferring resistance to diverse powdery mildew species^9^,^10^. The second involves *Mlo*, a widely conserved plant gene that negatively regulates plant defence responses and might be regarded as a powdery mildew susceptibility gene^11^,^12^. Recessive mutations in *Mlo* confer durable resistance to all races of *Bgh*. Such mutations were originally discovered in barley as artificial mutants from the 1940’s onwards and subsequently as a spontaneously occurring mutant in accessions collected in Ethiopia in the late 1930’s, designated *mlo-11*^13^. *mlo* alleles act early during infection and are characterised by rapid formation of large cell wall appositions (CWA) that correlate with resistance in epidermal cells, although a direct connection has not been demonstrated^14^.

*mlo* mutant based resistance has proved robust since first incorporated into European spring barley cultivars over thirty years ago^13^ and in Europe the naturally occurring *mlo-11* domain is the most widely used form of this resistance. *mlo-11* possesses a normal *Mlo* gene, with resistance conditioned by an upstream tandem repeat array^15^,^16^. This repeat array consists of copies of 5′ *Mlo* regulatory sequence and the first five exons of the *Mlo* gene, with the structure suggestive of ‘rolling-circle’- DNA replication used by plant viruses and transposons. Piffanelli *et al*.^15^ proposed a resistance mechanism involving aberrant transcriptional read-through from the upstream repeats supressing *Mlo* transcription.

Despite widespread deployment, *mlo* mutants are not without pleiotropic effects. Both induced mutants and *mlo-11* exhibit spontaneous necrosis that share a common QTL co-localising with *mlo*^17^. Excessive cell death in the absence of any pathogen leads to necrotic leaf spotting, associated loss of photosynthetic area and a reduction in yield^18^,^19^. Breeding efforts in Europe have combined *mlo* with genes that compensate for these effects but it has been implicated in increased susceptibility to facultative diseases such as spot blotch^20^ and Ramularia leaf spot^21^, although in the latter disease progression may be affected by environmental conditions^21^,^22^.

In the process of screening for new sources of resistance to powdery mildew, we uncovered a new variant of *mlo-11*. This variant, designated *mlo-11* (*cnv2*), exhibits partial resistance to powdery mildew in seedling leaves and effective resistance in adult leaves, without the pleiotropic effects of existing *mlo* alleles. In this study we investigate the phenotypic, structural, gene expression and epigenetic differences of the variant compared to cultivars possessing the wild-type *Mlo* gene or the standard *mlo-11* domain.

## Results

### Macroscopic Eth295 powdery mildew symptoms

**Figure 1:**
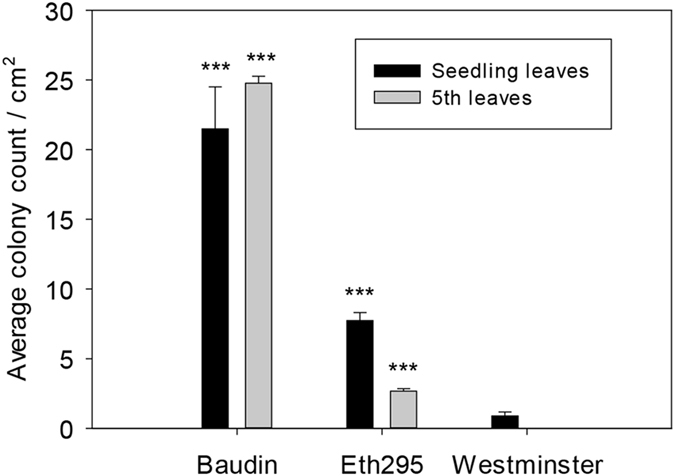
Macroscopic detached leaf *Bgh* powdery mildew colony counts. The graph depicts colonies per cm^2^ for barley cv. Baudin, landrace Eth295, and cv. Westminster at 7 dpi for seedling and fifth barley leaves inoculated with *Bgh* isolate Wag-001. Error bars are standard errors for five biological replicates per experiment with each experiment repeated three times. Significant differences were determined using the Student’s t-test, ***P < 0.001.

In experiments using single Australian *Bgh* isolates, Art-001, Wag-001 and Will-005, landrace Eth295 developed fewer colonies in detached leaves which progressed more slowly than the susceptible control cultivar (cv.) Baudin, with an infection type (IT) 2–3 at 7 dpi (see Fig. 1 and Supplementary Fig. 1 for isolate Wag-001) based on the scale of Kølster *et al*.^23^. A standard *mlo-11* control cultivar, Westminster, was resistant and showed water soaked lesions, suggesting underlying or mesophyll cell death, with occasional colonies stemming from infection through stomatal cells. Detached seedling leaf inoculations gave an average ratio of Baudin to Eth295 colonies of 6.5, with a large sample standard deviation across 3 isolates, s = 4.30. In seedling tests at IPK Gatersleben six isolates produced no symptoms and two (CH4.8 and D4/6) very few colonies (data not shown) and therefore further phenotypic characterisation of adult leaves was not conducted. In detached fifth leaf inoculations, Eth295 showed a consistently more resistant phenotype than Baudin by a factor of 11.28, s = 0.31.

**Figure 2:**
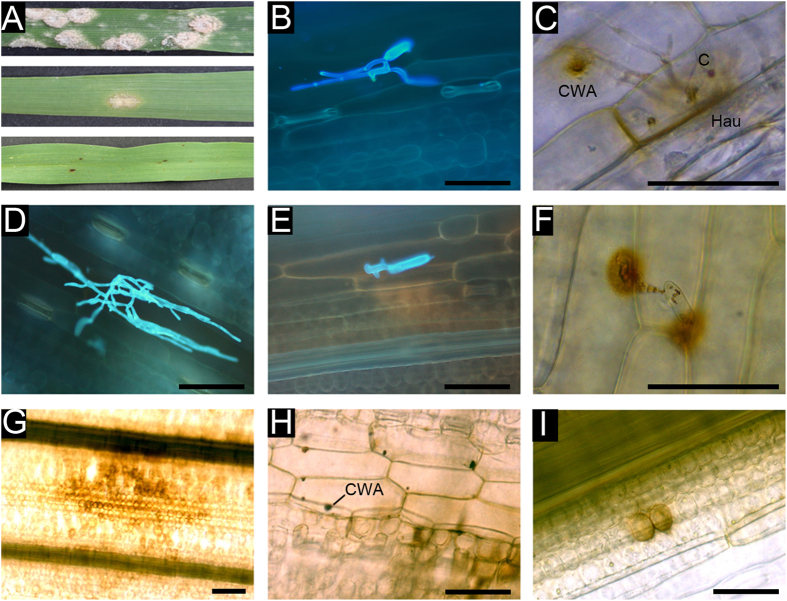
*Bgh* disease symptoms on barley leaves inoculated with *Bgh* isolate Wag-001. Figure (**A**) Whole plant macroscopic symptoms at 14 dpi on (from top to bottom) cv. Baudin, landrace Eth295 and, cv. Westminster. Figure (**B**–**F**) Microscopic *Bgh* disease symptoms on detached barley fifth leaves at 48 hpi inoculated with isolate Wag-001. B) Eth295 depicting limited secondary hyphal growth. (**C**) Eth295 illustrating a secondary cell wall apposition (CWA), haustorium (Hau) and conidium (**C**). (**D**) Baudin with rapidly developing hyphae. (**E**) Germinating conidium on Westminster with secondary and tertiary appressorial lobes. (**F**) Westminster, showing CWA restricted to epidermal cells immediately below a germinating conidium. Figure (**G–I**) Microscopic mesophyll cell death on uninfected detached barley fifth leaves. (**G**) Westminster stained with DAB showing the accumulation of hydrogen peroxide in numerous mesophyll cells. (**H**) Spontaneous CWA in cv. Westminster not observed in landrace Eth295. (**I**) Eth295 depicting hydrogen peroxide restricted to a few cells, in this example two cells. Samples (**B**,**C**,**E**) were triple stained with Evan’s blue, aniline blue and calciflour white. Samples D and F-I were DAB stained. Scale bars indicate 200 μm.

Whole plant Eth295 assays inoculated at the 5^th^ leaf stage showed fewer colonies compared to detached leaves. For example, at 14 dpi only three colonies were observed on eight Eth295 plants, while Baudin controls showed abundant sporulation, IT 4, with >50% of the leaf area infected (Fig. 2A).

### Cytological features of Eth295

Microscopic examination of 5^th^ leaf 48 hpi showed unsuccessful spore penetration rates of the epidermal cell wall between cv. Westminster, landrace Eth295, and cv. Baudin were ≥99%, 85–90%, and 25–30%, respectively. In Eth295, successful penetration was accompanied by secondary hyphae elongation (Fig. 2B,C). In general landrace Eth295 showed no auto-fluorescence beneath epidermal cells indicating a lack of mesophyll cell death compared to Westminster, while growth was limited compared to Baudin (Fig. 2D). In Westminster, fungal germlings produced an appressorial germ tube and attempted penetration from an initial appressorial lobe failed to penetrate the epidermal call, leading to second and third unsuccessful lobes (Fig. 2E) which are a feature of *mlo* based resistance^24^.

DAB staining of leaves 48 hpi was performed to detect CWA (rounded protuberances) and accumulation of hydrogen peroxide (H_2_O_2_), often a precursor of cell death. No epidermal accumulation of H_2_O_2_ was evident in Eth295 at attempted penetration sites, a feature shared with standard *mlo-11* cultivars (Westminster, Grange and Henley). Where penetration was successful, Eth295 showed secondary CWA in surrounding cells (Fig. 2C). Mesophyll accumulation of H_2_O_2_ was rarely observed, typically involving 2–8 superficial mesophyll cells. In Westminster, areas of mesophyll accumulation of H_2_O_2_ were large and more numerous than Eth295, typically involving 50 or more cells when viewed from above, while trypan blue staining confirmed extensive cell death throughout the mesophyll layer (Supplementary Fig. 1E,F).

Uninfected 5^th^ leaf segments of cv. Westminster *mlo-11* also exhibited areas of accumulation of H_2_O_2_ (in the range of 10–30 cells, Fig. 2G) while spontaneous papillae were also observed in Westminster and not in Eth295 (Fig. 2H). In Eth295, accumulation was rarely observed and confined to 2–4 superficial mesophyll cells (Fig. 2H). Cell death sites occurred on average at 31 sites per cm^2^ in Westminster compared to 4 sites for Eth295 (Supplementary Fig. 1G).

### Involvement of *mlo* in Eth295 resistance

Landrace Eth295 was crossed with cv. Baudin to investigate the genetics of *Bgh* resistance. All F_1_ progeny were fully susceptible to *Bgh*. One hundred and seventeen F_2_ lines were inoculated to determine the resistance segregation. Twenty seven individuals were resistant and 91 susceptible, indicating a 1:3 ratio. Chi squared was 2.67, *p* > 0.01, for the null hypothesis of no difference between the observed and expected segregation controlled by a single recessive locus. No F_2_ individuals showed disease symptoms intermediate to the parental phenotypes.

**Figure 3:**
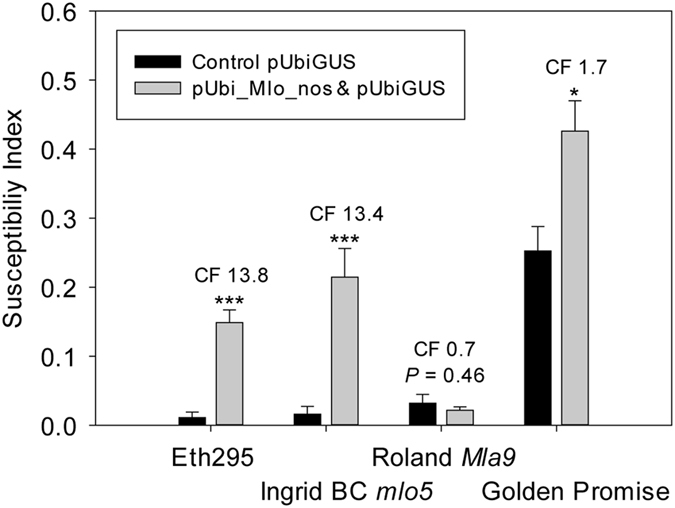
Biolistic complementation of *mlo* alleles with a construct overexpressing the *Mlo* gene. The graph depicts epidermal cell susceptibility indices for barley lines bombarded with pUbiGUS and pUbi_Mlo_nos. Control samples were bombarded with pUbiGUS only. Haustoria were counted 48 hr after inoculation with Swiss field isolate CH4.8 *avrMla9* by light microscopy. The graph compares results from barley landrace Eth295 with control genotypes cv. Ingrid *mlo5*, cv. Roland *Mla9*, and cv. Golden Promise 2012 *Mlo*. The susceptibility index was calculated as (ΣGUS-stained epidermal cells containing at least one haustorium/ΣGUS-stained epidermal cells). The complementation factor (CF, the ratio of the means of total haustoria in pUbi_Mlo_nos bombarded samples to the control samples) is given for each treatment pair. Error bars are standard errors based on using seven leaf segments per complementation experiment and each experiment was replicated a minimum of four times. Significant differences were determined using the Student’s t-test; ***P < 0.0005, and *P < 0.05.

The best known recessive resistance in barley is *mlo* conditioned, and therefore this locus was studied to establish any contribution to the Eth295 phenotype. Transient complementation with overexpressed *Mlo* was carried out by bombarding pUbi_Mlo_nos into epidermal leaf cells, followed by inoculation with *Bgh*. Both Eth295 and the control line cv. Ingrid BC *mlo5* showed an increased susceptibility index by a factor >5 (Fig. 3), which is indicative of *mlo* mediated resistance^4^. A slight increase in susceptibility was observed in the negative control cv. Golden Promise. This corresponds to the average effect found in a population of non-*mlo* accessions^4^ and may be regarded as a marginal gain in susceptibility from over-expressing *Mlo*.

As *mlo-11* is a known naturally occurring form of *mlo*, we conducted initial PCR experiments using two diagnostic genetic markers designed against the *mlo-11* domain^15^. These were inconclusive: The first primer pair amplifies a stowaway MITE located immediately 5′ to the *mlo-11* repeats. Eth295 exhibited a short wild-type amplicon of 381 bp, rather than the 441 bp in cv. Westminster (Supplementary Fig. 2). The second, targeting the *mlo-11* repeat units, showed weak and inconsistent amplification. The forward primer used to amplify this repeat, ADUP7, possesses a homopolymeric 3′ end. Non-specific amplification was excluded by amplicon sequencing which indicated at least one copy of the *mlo-11* repeat unit was present.

To resolve the number of *mlo-11* repeat units in landrace Eth295, new primers were developed that targeted *Mlo* exon 5 and digital PCR conducted to determine copy number variation (CNV). Digital PCR is a highly sensitive method for absolute quantification of nucleic acids without the need for standard curves. The PCR sample is partitioned into thousands of droplets, with each droplet containing a target sequence detected by fluorescence. Digital PCR is more accurate than conventional real-time qPCR which relies on relative quantification, while variations in amplification efficiency and signal saturation may affect results. The results indicated Eth295 contained two repeat units and the negative control Baudin no repeat units. Westminster displayed a high copy number. Despite 32 separate measurements, the number was only reliably estimated at 11–12 repeats (average = 11.92, s = 0.44).

To test whether the *mlo-11* repeat region and the *Mlo* locus was intact in Eth295, genetic linkage between a MITE SNP (Supplementary Fig. 2) and the resistance locus was examined. Only one recombinant was detected in 117 F_2_ progeny. To confirm *mlo-11* repeat subunit mediated resistance is cis-regulatory in Eth295, rather than a result of excision and reinsertion at a different site, PCR targeting read-through transcript from the *mlo-11* repeat units to *Mlo* exon 6 was positive (image not shown). No microsatellites 3′ to *Mlo* (HVMLOH1A, GBM1448, WMS6, HVM67, GBM1388 and GBM1324) were polymorphic between Eth295 and Westminster, except Bmag0138b, which lies >25 cM from the *Mlo* locus^19^,^25^.

### Intermediate levels of *Mlo* and *mlo-11* aberrant transcript expression in landrace Eth295

Expression data revealed landrace Eth295 expressed more than double the amount of aberrant transcripts at first and fifth leaf stages compared to cv. Westminster (Supplementary Fig. 4). Expression levels in cv. Baudin were zero; consistent with this cultivar lacking *mlo-11* repeats. Total *Mlo* transcripts were over three times higher in Eth295 than Westminster in seedling leaves and five times higher in fifth leaves. However, Westminster total *Mlo* transcripts were composed of entirely of aberrant transcript, with little or no normal *Mlo* transcription.

**Figure 4:**
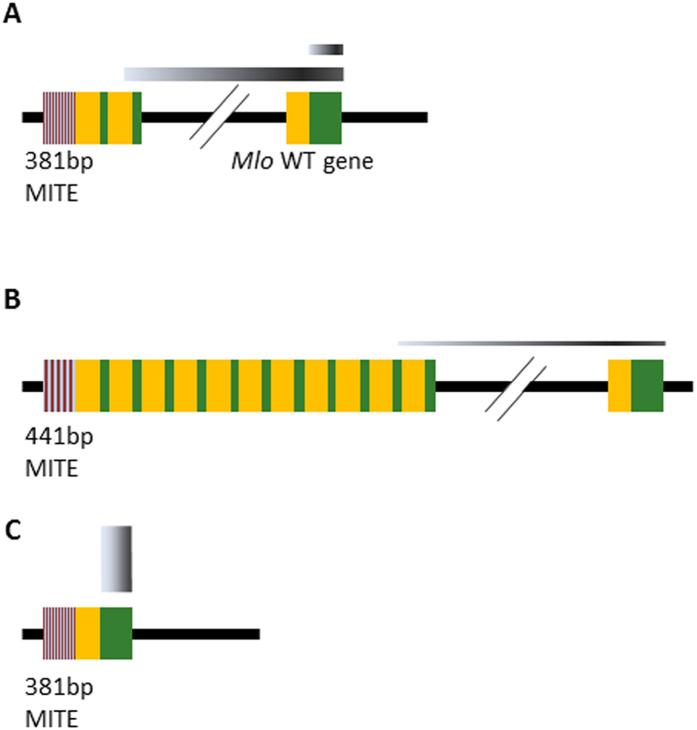
Schematic representations of domain organisation and transcript expression levels in *Mlo-11* variants. (**A**) Landrace Eth295. (**B**) Cultivar Westminster. (**C**) Wild type *Mlo* gene in *Bgh* susceptible cv. Baudin for comparison. Grey bar widths indicate relative expression levels of aberrant transcripts between the truncated *mlo-11* repeats and *Mlo* and of *Mlo* itself (see Supplementary Fig. 4). Yellow rectangles represent the promoter region of *Mlo* and green rectangles represent merged exons. The *mlo-11* arrangement in B is based on Peterhansel *et al*.^16^. DNA regions not drawn to scale. The line break indicates the exact distance between the 3′-proximal repeat unit and the *Mlo* wild-type gene copy is not resolved.

At the first leaf stage, Eth295 expression of normal *Mlo* transcript was less than half the level of Baudin, once aberrant *mlo-11* repeat transcripts are taken into account. At fifth leaf stage, Eth 295 expressed one fifth of the amount of *Mlo* compared to Baudin. These relative amounts are consistent with lower colony counts at the fifth leaf stage in Eth295. Figure 4 depicts the organisation and relative expression levels of Eth295, Westminster and Baudin *Mlo* and *mlo-11* repeat unit loci.

### Expression of *Mlo* is DNA methylation dependent

**Figure 5:**
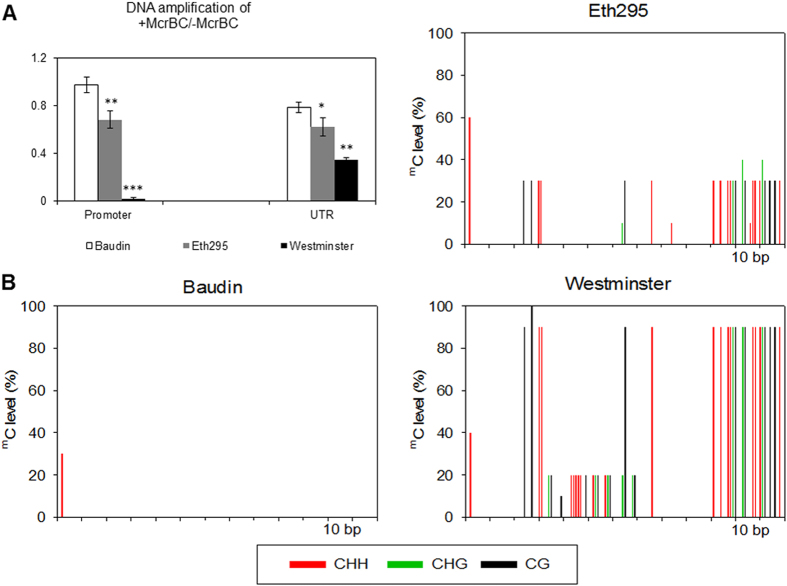
DNA methylation status of *Mlo* in landrace Eth295, cv. Baudin and cv. Westminster. (**A**) DNA methylation status was determined by digestion with McrBC followed by quantitative PCR at the promoter and UTR region of *Mlo* gene. Undigested genomic DNA was used as a control. Four biological replicates were used for each accession and real time PCR was performed with two McrBC digest replicates and four qPCR technical replicates. Error bars are standard errors and significant differences are determined using the Student’s t-test; ***P < 0.001, **P < 0.01 and *P < 0.05. (**B**) Methylation status of cytosine residues determined by bisulphite sequencing of a section of the promoter region of *Mlo* (−440 to −390 bp from the start codon). The percentage of ^m^C in three contexts (CG, CHG and CHH) was calculated by sequencing of eighteen clones from each accession.

DNA methylation levels of both the *Mlo* promoter and 5′ UTR regions were studied to examine methylation dependency. The restriction enzyme McrBC was used as a sensitive methylation detection method, rather than conventional methylation-dependent endonucleases. McrBC detects a high proportion of methyl cytosines by recognition of two short half sites with the consensus sequence (G/A)^m^C and, in addition, restricts asymmetric and hemi-methylated (single stranded) sites^26^. Digestion with McrBC followed by qPCR showed that cv. Westminster (together with cvs. Grange and Henley, data not shown) exhibited low qPCR amplification levels compared to Eth295 and non-*mlo* cv. Baudin at both read-through promoter sequences and 5′ UTR regions, indicating these regions are highly methylated. No evidence for methylation at the control actin promoter was found. Westminster methylation was strongest at the *Mlo* promoter (Fig. 5), resulting in negligible amplification (∆∆Cq = 0.008, s = 0.003). Landrace Eth295 displayed methylation levels intermediate between Westminster and Baudin, consistent with transcript levels at the 5′ UTR and the intron-spanning section of *Mlo* exons 6 and 7. Bisulphite sequencing is a process that involves treatment of genomic DNA to convert cytosine residues to uracil, but leaves 5-methylcytosine residues unaffected. Bisulphite sequencing of the promoter region also showed a higher level of DNA methylation and total number of methylated cytosine bases in Westminster, a low level in Baudin and an intermediate level in Eth295 for all the CG, CHG and CHH contexts (where H = A, T, or C, Fig. 5).

## Discussion

In this study we demonstrated Ethiopian landrace Eth295 possesses effective broad spectrum resistance to *Bgh* in adult leaves. Subsequent genetic analysis and complementation with overexpressed *Mlo* implicated a recessive *mlo* allele as underlying the resistance. Unlike known *mlo* alleles, the Eth295 variant does not show the pleiotropic effects of spontaneous necrosis and loss of photosynthetic tissue.

The resistance in seedling Eth295 leaves is quantitative in both the number of colonies and in the extent of colony growth. In detached leaf experiments, leaves exhibited proportionally higher numbers of powdery mildew colonies than adult fifth leaves. This is consistent with developmental control of spontaneous *mlo* CWA described by Wolter *et al*.^27^. However, Eth295 does show higher levels of infection than standard *mlo* cultivars, where colony development is only occasionally seen as a result of infection of stomatal subsidiary cells^28^ or as a result of drought-relief stress related to genetic background^29^. Notably, Eth295 is more resistant in intact (whole plant) assays than in severed leaves inoculated *in-vitro*. As *mlo* resistance is associated with physiological processes that prevent successful appressorial penetration of host epidermal cells, the condition of tissues in detached leaves may affect the timing and success of the resistance response.

Cytological studies showed Eth295 exhibits penetration resistance in common with standard *mlo-11*, represented in this study by the cultivar Westminster, with CWA observed below attempted penetration sites. Principal differences were presence in Eth295 of CWA in adjacent epidermal cells where penetration was successful at the initial site. Extensive necrosis of surrounding cells and the collapse of underlying mesophyll cells were not observed in Eth295. Similarly, in uninfected leaves, Eth295 exhibited no spontaneous CWA compared to Westminster, and limited spontaneous necrosis, typically restricted to two to four cells. Partial seedling resistance similar to Eth295 has been previously observed in chemically induced *mlo-12* & *mlo-28* mutants of *Mlo* wild-type plants^30^. Infected leaves indicated this resistance was accompanied by lower levels of mesophyll cell death than other *mlo* alleles. However, moderate cell death was still evident and contrasts with Eth295. The phenotype of Eth295 supports the hypothesis of Buschges *et al*.^12^ that the *Mlo* protein negatively regulates multiple defence-related processes with the severity of the phenotype dependent on the strength of the mutation in inhibiting Mlo protein formation.

We examined the structure and gene expression levels of the *mlo-11* subunit domains and the *Mlo* gene itself in Eth295 and cv. Westminster to determine whether any variances might explain their phenotypic differences. Digital PCR was conducted to determine copy number variation (CNV), rather than qPCR as imperfect amplification efficiencies and signal saturation limits CNV measurement in samples with high copy numbers. This revealed a six-fold difference between Eth295 and Westminster, with 2 copies in Eth295 and 11–12 in Westminster. The copy number in Westminster could not be more precisely fixed as such CNV counts represent the limit of whole number precision for this technique^31^.

Eth295 contains a distinctive short MITE amplicon compared to Westminster and other *mlo-11* lines but no *mlo-11* repeat unit SNPs were detected between Eth295 and cv. Westminster. Together with a lack of polymorphic microsatellites immediately downstream from *Mlo*, a recent shared ancestry rather than an independent origin appears likely. Eth295 contains a SNP shared with Westminster at the 3′ end of the MITE containing amplicon. The region immediately 5′ to this SNP does not align between short and long versions of the amplicon due to an insertion consisting of contiguous sections of *Mlo* exon 5 and intron 5 (Supplementary Fig. 2). The shared SNP and short MITE containing amplicon in Eth295 may be explained by unequal crossing over and gene conversion between progenitor repeat units or recombination between these repeat units and the wild-type *Mlo* gene during meiosis, followed by a second recombination event with a wild type *Mlo* line MITE region. However, the most parsimonious explanation for the Eth295 arrangement is direct recombination just before the shared SNP with a homologous 5′ promoter region present between the *mlo-11* repeat units.

In this study, relative *Mlo* expression levels between Eth295 and cv. Westminster corresponded to phenotypic differences. Piffanelli *et al*.^15^ were unable to detect an involvement of DNA methylation in gene expression and suggested read-through transcripts from *mlo-11* repeats, which lack a transcription termination signal, interfere with transcription from the downstream native *Mlo* promoter. However, in Eth295 *mlo-11* read-through transcripts and *Mlo* itself are expressed at twice the levels found in Westminster, which showed no *Mlo* gene expression. Disruption of transcription machinery assembly by such read-through is therefore unlikely to be a major mechanism controlling *Mlo* expression in *mlo-11*.

Methylation of repetitive DNA is a common plant defence mechanism against viruses and transposons^32^. The involvement of methylation in the expression of the *Mlo* gene conditioned by different *mlo-11* subunit domains was shown by qPCR following restriction with McrBC and direct detection of methylation at the *Mlo* promoter by bisulphite sequencing. These corresponded to intermediate *Mlo* qPCR transcript levels in landrace Eth295 and no *Mlo* expression in cv. Westminster, indicating a direct relationship with *mlo-11* repeat unit copy number. As repetitive DNA is associated with the formation of double stranded RNA leading to RNA-directed methylation via small interfering RNAs, future analyses may elucidate the role of that process and of different histone modification pathways in *mlo-11* epigenetic regulation^33^,^34^,^35^.

Landrace Eth295 is a selection of HOR 2543 (*Hordeum vulgare* L. convar. *deficiens* var. *nudideficiens* Körn.), collected by the ‘Zentralinstitut für Genetik und Kulturpflanzenforschung Gatersleben’ in Germany. No collection information is available in public databases for Eth295, however it is plausible to speculate this landrace may have arisen by natural selection against the pleiotropic effects of standard *mlo-11*. The existence of *mlo-11* (*cnv2*) suggests rare *mlo-11* repeat copy number recombinants offer breeders an opportunity to fine tune the balance between copy number, pleiotropic effects and resistance levels, allowing the incorporation of such alleles into cultivars without selecting genetic backgrounds that compensate for the pleiotropic effects.

## Methods

### *Bgh* maintenance and plant growth

Mono-conidial Australian *Bgh* cultures from Australian field isolates were produced and pathotyped as described by Tucker *et al*.^36^. Powdery mildew isolates were maintained and propagated on detached leaf sections of cv. Baudin inserted into 50 mgL^−1^ benzimidazole agar plates and grown in a Contherm Biosym 6200CP4 incubator (16 °C and 10 °C under a 12 h light and dark cycle, respectively). Isolates were sub-cultured at 10 days or before leaves senesced.

Barley plants for seedling leaf detached leaf infection assays were grown in vermiculite fertilised with Nitrophoska Perfect (EuroChem Antwerpen NV, Belgium) and under light shelves at 300 μmol m^−2^ s^–1^. Plants for detached 5^th^ leaf or whole plant infection assays were grown in soil with Nitrophoska Perfect in a controlled temperature room (18–22 °C) and a 12 h photoperiod at 450 μmol m^−2^ s^–1^.

### Detached leaf and whole plant disease assays

For all experiments, a minimum of five biological replicates were used and the experiments repeated three times. Landrace Eth295 was obtained from the Australian Grains Genebank (Horsham, VIC) and was identified as having prominent adult plant-like resistance in primary disease screens against Australian isolates. Cultivar Westminster was used as a standard *mlo-11* control with cvs. Grange and Henley as additional comparative replicates (Limagrain-Nickerson, UK). Cultivar Baudin was used as a positive control. All experiments in Western Australia were conducted with isolate Wag-001. Two different pathotypes, Art-001 and Will-005 were used in comparative replicate experiments. Isolates CH4.8, RiIII, Ro93a, MH21, D35/3, D35/2, D4/6, D2/4 were used for inoculations at IPK Gatersleben. Detached leaves were inoculated as described by Tucker *et al*.^36^. Whole plant inoculations at the fifth leaf stage were performed by placing plants into trays enclosed in 80 cm high polythene tents, and spores allowed to settle from above the tents. Inoculated plants were sealed and kept within the polythene tents for 24 hr then placed in a controlled temperature glasshouse with a maximum temperature 22–24 °C.

Inoculated detached leaves were scored macroscopically at 7 days post inoculation (dpi), unless otherwise indicated and rescored at 10 dpi to confirm more resistant disease reactions. A five point scale was used based on that devised based on Mains *et al*.^37^ and Kølster *et al*.^23^, where 0 = no visible mycelium, 1 = sparse mycelial development with no sporulation, 2 = mycelial present with very few spore chains, 3 = moderate mycelial development, discrete lesions with sporulation, and 4 = amorphous mycelial development and abundant sporulation. Colony counts were obtained from 2 cm wide transects across detached leaf plates.

### Cytology

*Bgh* infected leaves were sampled at 48 hpi by taking 0.5 cm^2^ leaf segments from 10 independent leaves per genotype. A modified 3,3-diaminobenzidine (DAB) uptake was carried out as described by Thordal-Christensen *et al*.^38^. Triple staining with Evans blue, aniline blue and calcofluor white was performed following the protocol developed by Felix Mauch’s Group at the University of Fribourg^39^. Trypan blue retention on 0.3 mm wide mesophyll cross sections was performed as described by van Wees^40^ using Farmer’s fluid (acetic acid: ethanol: chloroform at 1:6:3) rather than chloral hydrate solution to de-stain the tissue. DAB staining of mesophyll cells was performed on epidermal strips as described by Thordal-Christensen *et al*.^38^.

### Genetic analysis

Dominance of the Eth295 resistance phenotype was determined among five F_1_ Baudin x Eth295 progeny by inoculating detached 1^st^ and 5^th^ leaves. Whole plant F_2_ progeny were inoculated at the 5^th^ leaf. To confirm the location of the resistance locus (also see *mlo* complementation below) F_2_ genotyping was performed with the nearest *Mlo* genetic marker. This was a unique Eth295/Baudin T/C SNP within the MITE at position 7659 relative to GenBank accession Y14573 (Supplementary Fig. 2) amplified by primers Mlo6 and Mlo10 described by Piffanelli *et al*.^15^. This polymorphism served as a CAPS marker for PCR products following restriction with *Hinf*I and resolution on a 2.5% agarose gel.

### *mlo* complementation

Leaf segments of accession Eth295 were co-bombarded with a mixture of GUS- reporter plasmid pUbiGUS^41^ and pUbi_Mlo_nos carrying wild type *Mlo*^12^ as described by Spies *et al*.^4^. Ingrid BC *mlo*5 was used as a positive *mlo* control; cvs. Golden Promise and Roland *Mla9* were used as negative *Mlo* controls. Each accession was bombarded with or without pUbi_Mlo_nos, using 7 leaf segments per treatment and each experiment replicated a minimum of four times. Four hours after bombardment leaf segments were inoculated with *Bgh* isolate CH4.8 *avrMla9* at a density of approx. 150 conidia/mm^2^, and haustoria in GUS stained (transformed) epidermal cells counted 48 h after inoculation. The susceptibility index (SI) was calculated as (ΣGUS-stained epidermal cells containing at least one haustorium/ΣGUS-stained epidermal cells).

### Eth295 *mlo-11* locus structure

The *mlo-11 locus* was initially examined by PCR with previously published MITE and *mlo-11* repeat primer pairs, Mlo6-Mlo10 and ADUP7-Mlo6, respectively^15^. PCR products were run out on a 1.5% agarose gel and Sanger sequenced to identify nucleotide differences. The following microsatellites were tested for polymorphism between Eth295 and Westminster 3′ to the Mlo gene: HVMLOH1A, GBM1448, WMS6, HVM67, GBM1388, GBM1324, and Bmag0138b^19^,^25^.

Copy number variation (CNV) of landrace Eth295 was determined with QuantStudio™ 3D Digital PCR System (Life Technologies, Carlsbad, CA, USA). The *mlo-11* cultivar Westminster was used as the standard *mlo-11* domain control, and cv. Baudin as a wild-type *Mlo* control. Cultivars Grange and Henley provided additional standard *mlo-11* domain controls. Sample DNA concentrations were measured with a Quibit 2.0 Fluorometer (Life Technologies). Primers MloEx5_F and MloEx5_R were designed to *Mlo* exon 5, together with a VIC Taqman probe MloEx5T. These primers detect *mlo-11* subunits together with the WT *Mlo* gene. Reference single copy actin primers (designed from GenBank accessions AY145451 and CAJX010010156) were Actin F and Actin R, combined with a FAM labelled Taqman probe. DNA restriction with *MseI* was used to cleave linked copies of the target exon for dilution into separate PCR partitions. Digital PCR DNA partition, PCR amplification, chip reading and data analyses were performed according to manufacturer’s recommendations. For each accession, four biological replicates were used, with two *Mse*I digests per biological replicate, followed by four technical replicates per *Mse*I digest. To confirm read-through transcription between *Mlo-11* and *Mlo*, forward primer MloPro_F2 and reverse primer MloEx6_R were used in conventional PCR. These primers amplify a 1650 bp DNA region -227 bp relative to the *Mlo* transcriptional start site through to *Mlo* Exon 6. All primer sequences used in this study are provided in Supplementary Fig. 3 and illustrated with Geneious v. R8^42^.

### Gene expression

Four biological replicates of leaf tissue were sampled from leaves of each accession at the 1^st^ and 5^th^ leaf stages. Total RNA was extracted using TRIzol® Reagent (Life Technologies) following the manufacturer’s protocol. cDNA was synthesised from 3 ug total RNA using a RNA QuantiTect Reverse Transcription Kit (Qiagen, Hilden, Germany). Quantitative real-time PCR (qPCR) was performed on a C1000TM Thermal Cycler (Bio-Rad, Hercules, CA, USA) with SYBR green detection (QuantiTect SYBR Green PCR Kit) with three technical replicates. Primers were developed to target *mlo-11* aberrant transcripts only by amplifying the *mlo-11* repeat region read-through promoter sequence with the primers MloPro_F3 and MloPro_R1. These amplify a 138 bp section upstream of the *Mlo* 5′ UTR. A second set of primers amplified total *Mlo* (*Mlo* and *mlo-11* read-through) transcripts from an intron-spanning section of *Mlo* exons 6 and 7, as the *mlo-11* repeat units are composed of *Mlo* exons one to five^15^. These transcripts were detected with the primers MloEx6_F and MloEx7_R, which amplify a 100 bp region. Internal reference primers used were Actin F and R, as described for CNV above. Relative transcript levels were calculated by the ∆∆Ct method factoring in primer efficiencies.

### Cytosine methylation by McrBC-quantitative PCR

Methylation of the promoter region of *Mlo* and the *mlo-11* repeat units were detected by McrBC (NEB, Ipswich, MA) digestion followed by qRT-PCR. McrBC detects a high proportion of methyl cytosines by recognition of two short half sites with the consensus sequence (G/A)^m^C and, in addition, restricts asymmetric and hemi-methylated (single stranded) sites^26^. For each accession, two 500 ng aliquots of DNA from four biological replicates were treated with McrBC. Undigested controls were treated with water. qPCR was performed with four technical replicates per sample and DNA methylation levels quantitatively calculated by the ∆∆Ct method. Detection primers for *Mlo* and *mlo-11* 5′ UTR were forward primer MloUTR_F and reverse primer MloUTR_R. These primers amplify a 234 bp DNA region. Primers for the promoter region before the 5′ UTR were forward primer MloPro_F1 and reverse primer MloPro_R1. These primers amplify a 311 bp DNA region. Primers for the actin promoter region were forward primer ActPro_F and reverse primer ActPro_R, which amplify a 297 bp DNA region. As the *Mlo* promoter and 5′ UTR sequences are also identical in all *mlo-11* repeat units, the qPCR results represent the overall (*Mlo* and *mlo-11*) methylation status.

### Cytosine methylation bisulphite sequencing analysis

Bisulphite sequencing was performed based on published protocols^34^,^35^ with genomic DNA extracted from landrace Eth295, cv. Baudin and cv. Westminster. 200 ng of each accession was subjected to bisulphite CT conversion using an EZ DNA Methylation-Direct kit (Zymo Research, Irvine, CA). Bisulphite converted DNA was amplified by PCR using primers BiSu_F and Bisu_R. Untreated DNA was used for control reactions. Amplified fragments were cloned into the pGEM-T vector (Promega, Madison, WI) and eighteen ligated clones from each accession sequenced.

## Additional Information

**How to cite this article**: Ge, X. *et al*. Tempered *mlo* broad-spectrum resistance to barley powdery mildew in an Ethiopian landrace. *Sci. Rep.* **6**, 29558; doi: 10.1038/srep29558 (2016).

## Supplementary Material

Supplementary Information
