# Supplementary material for: Tempered mlo broad-spectrum resistance to barley powdery mildew in an Ethiopian landrace
Source: Sci Rep. 2016 Jul 12;6:29558. doi: 10.1038/srep29558 (PMC4941727; doi:10.1038/srep29558)
Supplement: Supplementary Information [file srep29558-s1.doc]

Tempered *mlo* broad-spectrum resistance to barley powdery mildew in an Ethiopian landrace

Xintian Ge^1#^, Weiwei Deng^1#^, Zheng Zhou Lee^1^, Francisco J. Lopez-Ruiz^1^, Patrick Schweizer^2^ and Simon R. Ellwood^1*^

^1^ Centre for Crop and Disease Management, Department of Environment and Agriculture, Curtin University, Bentley, WA 6102, Australia

^2^ Leibniz-Institut für Pflanzengenetik und Kulturpflanzenforschung (IPK) Gatersleben, Corrensstrasse 3, Seeland, 06466 Stadt, Germany

^#^ These authors contributed equally

Supplementary Figures

Supplementary Figure 1: P2

Supplementary Figure 2: P3

Supplementary Figure 3: P4-5

Supplementary Figure 4: P6


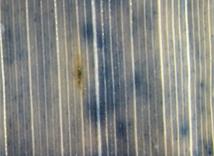

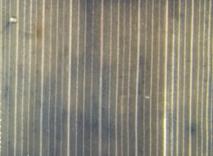

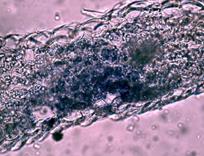

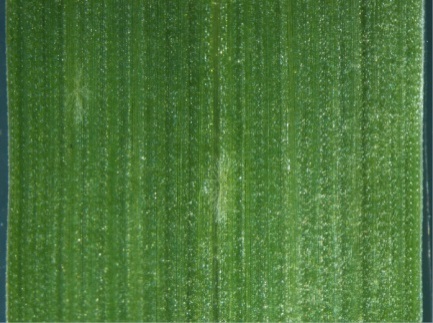

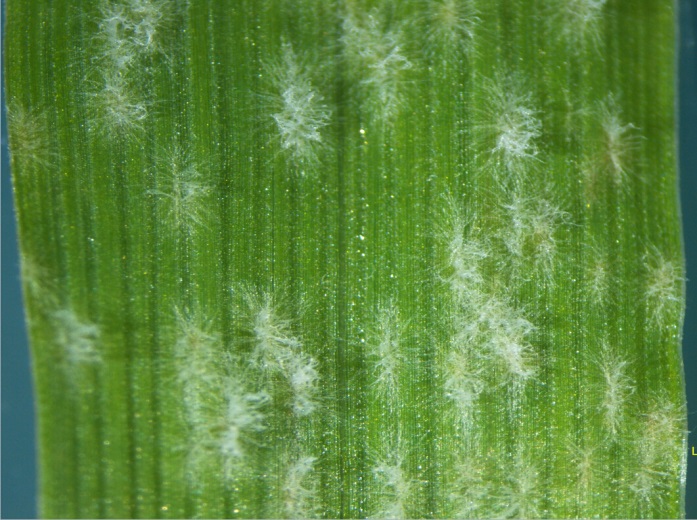

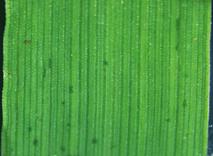


**A**

**B**

**C**

**D**

**E**

**F**

**G**

**Supplementary Figure 1.** A-E: Macroscopic *Bgh* disease symptoms on barley leaves inoculated with *Bgh* isolate Wag-001. A, B, and C: Fifth leaves at 7 dpi showing cv. Baudin, landrace Eth295, and cv. Westminster, respectively. Note water soaked lesions in Westminster. D-F: Trypan blue stained leaves 48 hpi. D) Eth295 with little apparent cell death. E) Westminster showing intense areas of trypan blue indicating cell death. F) Leaf cross section of Westminster showing underlying mesophyll cell death. Scale bar indicates 200 μm. G) Cell death sites per cm^2^ in uninfected Westminster adult fifth leaves, compared to Eth295 and Baudin. Error bars are standard errors for five biological replicates per experiment with each experiment repeated three times. Significant differences were determined using the Student’s t-test, *** = *P*<0.001. Note Westminster cell death sites are much larger than in Eth295 (see Results: Cytological features of Eth295).

Eth295 1 GTCCTGCCACCTAAGTAGCAGCCAAAGATGCAGTCCACGCTCCATTTGACTTGACTCGCT

Baudin GTCCTGCCACCTAAGTAGCAGCCAAAGATGCAGTCCACGCTCCATTTGACTTGACTCGCT

Wm GTCCTGCCACCTAAGTAGCAGCCAAAGATGCAGTCCACGCTCCATTTGACTTGACTCGCT

Eth295 61 CAGGCCCGTACTCCACCGGATGCAGACCACCGGCCGGCCACTTCGCCGGGACCCGCATGG

Baudin CAGGCCCGTACTCCACCGGATGCAGACCACCGGCCGGCCACTTCGCCGGGACCCGCATGG

Wm CAGGCCCGTACTCCACCGGATGCAGACCACCGGCCGGCCACTTCGCCGGGACCCGCATGG

Eth295 121 TCGACTTTTCCACGCTCGGGCGTCAAACTCGGAATGCCACGTCGACGTCCACGTCCTCGG

Baudin TCGACTTTTCCACGCTCGGGCGTCAAACTCGGAATGCCACGTCGACGTCCACGTCCTCGG

Wm TCGACTTTTCCACGCTCGGGCGTCAAACTCGGAATGCCACGTCGACGTCCACGTCCTCGG

Eth295 181 TCCGGTCGCATGCCGGCGTACGCGTGCCAGTCGCCACAACTTGCGTGCTACTTACAGTAC

Baudin TCCGGTCGCATGCCGGCGTACGCGTGCCAGTCGCCACAACTTGCGTGCTACTTACAGTAC

Wm TCCGGTCGCATGCCGGCGTACGCGTGCCAGTCGCCACAACTTGCGTGCTACTTACAGTAC

Eth295 241 GAGACTACTACTCCCTCGATTTGAAAATATTTATCCTAAAAATACATGTATCTAGACTTA

*Hinf*I

Baudin GAGACTACTACTCCCTCGATTCGAAAATATTTATCCTAAAAATACATGTATCTAGACTTA

Wm GAGACTACTACTCC----------------------------------------------

Eth295 301 TTTTATTTATAGATGTA-------------------------------------------

Baudin TTTTATTTATAGATGTA-------------------------------------------

Wm -----------------TACTACTTACAGTCGCCACCTGGGCCTCTCCAGCACCCCTGGC

3’ *Mlo* exon 5

Eth295 361 ------------------------------------------------------------

Baudin ------------------------------------------------------------

Wm ATCAGATGGGTGGTGAGTTTTTTAGCTTCTTATCTGCCCCTCATCTGTGTGTAATGTTTG

5’ *Mlo* intron 5

Eth295 421 --------------------TCTATTTTATTTATTTTTAGGATAACTATTTCTGGATGAA

Baudin --------------------TCTATTTTATTCATTTTTAGGATAACTATTTCTGGATGAA

Wm GCGTATGGAGTTAGGTGAGTTCTATTTTATTTATTTTTAGGATAACTATTTCTGGATGAA

Eth295 481 GGGGGTACATGCTAGTAGTAGATG

Baudin GGGGGTACATGCTAGTAGTAGATG

Wm GGGGGTACATGCTAGTAGTAGATG

**Supplementary Figure 2**. Alignment of barley stowaway MITE and adjacent *Mlo* 5’ promoter sequences between landrace Eth295, cv. Baudin and standard *mlo-11* cv. Westminster (Wm). Primers Mlo10 and Mlo6 were used to amplify the PCR products (Piffanelli *et al*., 2004). A unique Eth295 SNP is depicted at position 262. The second SNP at position 452 is located in the *Mlo* 5’ promoter sequence and shared with cv. Westminster. The 3’ *Mlo* exon 5 and 5’ *Mlo* intron 5 sequences present in the cv. Westminster MITE are indicated in blue and grey, respectively. *mlo-11* specific dinucleotides GT at the terminal end of the 5’ *Mlo* intron 5 sequence and which separate each repeat unit are highlighted in green. MITE amplicon sequences are deposited in GenBank under accessions KT873800-KT873802.


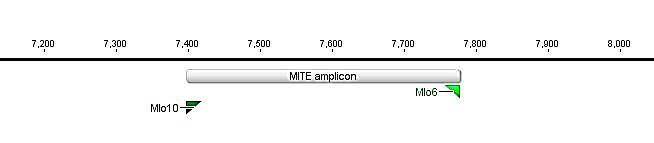


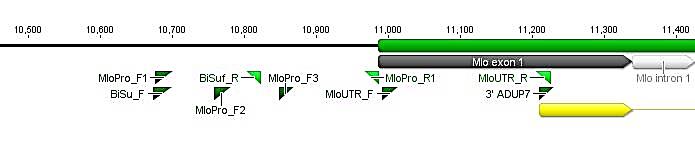


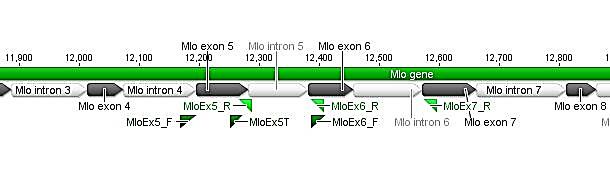


MloEx5_F TATGTTCATTTTTTGTGTCCTTTCAG

MloEx5_R AACTCACCACCCATCTGATGC

MloEx5T VIC Taqman probe CTCTCCAGCACCCCTGGCATC

Actin F TGTAAGGGACATCAAGGAGAAGC

Actin R GGAAGCTCGTAGCTCTTCTCAAC

Actin FAM Taqman probe CTTGGCAGTCTCCAGCTCCTGTTC

MloPro_F2 CCATGCCATCACTGAATCAAC

MloEx6_R ACCTGAAGAACTGCCTGAAGAAG

MloPro_F3 GGCTGTGTGTTTCGGGCTC

MloPro_R1 CGTGGCAAAGTGTGGCAAC

MloEx6_F TTCTTCAGGCAGTTCTTCAGGTC

MloEx7_R CTTGCTGTTTTGCGACAAATG

MloUTR_F TCCGCCAGCAAACCAGACAC

MloUTR_R CTTTTTTGTCCGACATCGGTC

MloPro_F1 GCAAGAAAGGAAAAGAAATAGTGC

ActinPro_F GCTGGAGTAGGTAATGCGTAAAATG

ActinPro_R CTCACCGTCAGCCATTTCCTAC

Bisu_F AGGYAAGAAAGGAAAAGAAATAGTG

Bisu_R CRACCRATRTTTTCATCCTA

**Supplementary Figure 3.** Sequences of primers used in this study and the positions of amplicon primers within and around the barely *Mlo* gene. All primer sequences are provided 5’-3’. Primer locations are aligned to GenBank accession Y14573 and displayed in Geneious v 8. Gene regions are shown in green, exons in dark grey, introns in light grey and coding sequences in yellow. Primers ADUP7, Mlo10 and Mlo6 were published by Piffanelli *et al*. (2004).

**A**

**B**

**Supplementary Figure 4.** Transcript expression levels of *Mlo* and read-through aberrant transcript, with landrace Eth295 compared to cv. Baudin (wild-type *Mlo*) and cv. Westminster (standard *mlo-11*). Expression levels on the y-axis are presented relative to actin as an internal control. A) Seedling expression depicting total transcript levels (at *Mlo* exons 6 and 7 and aberrant read-through transcript at the *Mlo* promoter) and aberrant transcript only. B) Fifth leaf expression also depicting total transcript levels and aberrant transcript only. Error bars are standard errors and are based on four biological replicates with three technical qPCR replicates. Significant differences were determined using the Student’s t-test, *** = *P*<0.001, **= *P*<0.01 and *= *P*<0.05.
